# *IL1B* Polymorphism (rs1143634) and IL-1β Plasma Concentration as Predictors of Nutritional Disorders and Prognostic Factors in Multiple Myeloma Patients

**DOI:** 10.3390/cancers16071263

**Published:** 2024-03-24

**Authors:** Marcin Mazurek, Aneta Szudy-Szczyrek, Iwona Homa-Mlak, Marek Hus, Teresa Małecka-Massalska, Radosław Mlak

**Affiliations:** 1 Department of Human Physiology of Chair of Preclinical Sciences, Medical University of Lublin, 20-080 Lublin, Poland; iwona.homa-mlak@umlub.pl (I.H.-M.); teresamaleckamassalska@umlub.pl (T.M.-M.); 2 Department of Haematooncology and Bone Marrow Transplantation, Medical University of Lublin, 20-080 Lublin, Poland; aneta.szudy-szczyrek@umlub.pl (A.S.-S.); markhus@o2.pl (M.H.); 3 Department of Laboratory Diagnostics, Medical University of Lublin, 20-080 Lublin, Poland; radoslawmlak@umlub.pl

**Keywords:** multiple myeloma, cancer cachexia, malnutrition, interleukin 1 beta, single-nucleotide polymorphism

## Abstract

**Simple Summary:**

According to the literature, 35–71% of multiple myeloma patients have nutritional disorders. In the development of cachexia and malnutrition, the inflammatory process, accompanied by an increase in the level of proinflammatory cytokines, plays a key role. Interleukin-1β is a cytokine that plays an important role in the mechanisms responsible for muscle and adipose tissue breakdown during malnutrition and cachexia. This study aimed to investigate the association of *IL1B* gene polymorphism and interleukin-1β plasma concentration with the occurrence of nutritional disorders and survival in patients with multiple myeloma. The presence of the CC genotype of the *IL1B* gene was associated with a higher plasma concentration of interleukin-1β, a higher risk of cachexia, and poor prognosis. Determination of *IL1B* polymorphism may be a useful predictive marker of the risk of cachexia and prognostic factor in multiple myeloma patients.

**Abstract:**

Background: Multiple myeloma (MM) is a hematological neoplasm of the early precursor of B-cells. The most characteristic symptoms observed during MM include hypocalcemia, anemia, bacterial infections, and renal damage. Nutritional disorders, especially malnutrition, are noted in about 35–71% of MM patients. Interleukin 1 beta (IL-1β) is a proinflammatory cytokine responsible for muscle atrophy and lipolysis during malnutrition and cachexia. This study aimed to evaluate the usefulness of the *IL1B* single-nucleotide polymorphism (SNP) (rs1143634) and plasma concentration of IL-1β in the assessment of the risk of nutritional disorders and prognosis in patients with MM. Methods: In our study, 93 patients with the de novo MM were enrolled. The real-time PCR with specific TaqMan probes method was used in genotyping. The IL-1β ELISA kit was used to determine IL-1β concentration in plasma samples. Results: Patients with the CC genotype, compared to the carriers of the other variants of the *IL1B*, demonstrated significantly higher concentrations of IL-1β in plasma (7.56 vs. 4.97 pg/mL), a significantly higher risk of cachexia (OR = 5.11), and a significantly higher risk of death (HR = 2.03). Moreover, high IL-1β plasma level was related to a significantly higher risk of cachexia (OR = 7.76); however, it was not significantly associated with progression-free survival (PFS) or overall survival (OS). Conclusions: Determination of the *IL1B* SNP (rs1143634) and plasma concentration of IL-1β may be useful in the assessment of the risk of cachexia and prognosis in patients with MM.

## 1. Introduction

Multiple myeloma (MM) is a hematological malignancy characterized by clonal proliferation of abnormal plasma cells in the bone marrow (BM). In highly developed countries, MM accounts for 1.3% of all cancers, and its incidence is 4.5–6/100,000 per year. MM is the second most common hematological cancer [1,2]. Moreover, MM accounts for 10% of all hematological malignancies [3]. MM most often affects patients over 65 years of age and is 1.5 times more likely to affect men [4,5]. In the course of MM, acute renal failure (19%), anemia (73%), hypercalcemia (15%), and osteolytic lesions are observed in bones (79%) (referred to as CRAB symptoms) [6]. The most common cytogenetic changes occurring in MM are del(1p), del(17p), del(13), gain(1q), t(4;14), t(14;20), and t(14;16) [3]. Frequently observed mutations include those located in *KRAS*, *NRAS*, *BRAF*, *EGR1,* and *FGFR3*. Moreover, in approximately 20% of MM cases, there are mutations in the nuclear factor kappa B (NFκB) pathway within the *TRAF3*, *NFKBIA*, *CYLD*, and *BIRC2/3* genes [7]. The basic method of treatment in MM is chemotherapy (CTH) based on the use of immunomodulatory drugs (IMIDs) such as thalidomide, lenalidomide, and bortezomib supplemented with autologous hematopoietic stem cell transplantation (aHSCT).

It is estimated that malnutrition occurs in up to 71% of patients with MM before treatment [8]. Moreover, malnutrition during treatment is observed in 35–45.4% of patients [9,10]. The risk factors of malnutrition/cachexia in cancer patients include more advanced disease stage, some tumor locations, more aggressive type of treatment, comorbidities, and low initial body weight [11]. Additionally, after aHSCT, patients are at risk of malnutrition due to CTH-related toxicity, higher risk of infection, and longer hospital stays [12]. Based on the guidelines proposed by the European Society of Clinical Nutrition and Metabolism (ESPEN), malnutrition was divided into three categories: disease-related malnutrition (DRM) with inflammation, DRM without inflammation, and malnutrition without comorbid disease [13]. Diagnostic criteria developed by the Global Leadership Initiative on Malnutrition (GLIM) include unintentional weight loss, low body mass index (BMI), reduced muscle mass, reduced food intake or absorption, and comorbid disease [14]. Cachexia is a multifactorial process that usually occurs in advanced cancer. Anorexia, anemia, muscle wasting, weight loss, and changes in protein, carbohydrate, and lipid metabolism are typical symptoms of cachexia [15,16]. Diagnostic criteria for cachexia include, in the case of cancer, a weight loss of at least 5% over 3–6 months or a BMI < 20 kg/m^2^ as the primary criterion [17], and, additionally, decreased muscle strength, fatigue, anemia, low free fat mass index (FFMI), decreased albumin levels, and increased proinflammatory cytokines, e.g., C-reactive protein. (e.g., CRP). Serum CRP level measurement is a useful marker in the diagnosis of cachexia. However, it should be noted that cachexia may occur without a systemic inflammatory process [18]. The occurrence of malnutrition/cachexia is associated with deterioration of quality of life, increased risk of infection, reduced response to treatment, and shortened overall survival (OS) of patients [19,20].

Thus far, the involvement of interleukin 1 beta (IL-1β) in the pathomechanism of MM has been demonstrated. IL-1β is a cytokine secreted by myeloma cells and it is responsible for bone resorption in MM. IL-1β stimulates stromal cells in the BM to secrete interleukin 6 (IL-6) [21,22]. Moreover, it has been shown that proinflammatory cytokines, including IL-1β, IL-6, interleukin 8 (IL-8), and tumor necrosis factor-alpha (TNF-α), are involved in the breakdown of fat and muscle tissue, which indicates their potential diagnostic usefulness in predicting, diagnosing, and monitoring cancer cachexia [23,24]. However, to date, there are no studies describing the relationship between IL-1β and the nutritional status of MM patients. On the other hand, it is involved in the induction of inflammation (in the hypothalamic–pituitary–adrenal axis) and the mechanisms responsible for the breakdown of muscle fibers and lipolysis [25]. Single-nucleotide polymorphism (SNP) rs11436349 (+3954C>T) of the *IL1B* gene is a silent polymorphism of exon 5 in chromosome 2. Some studies refer to its functional nature resulting in a higher IL-1β secretion [26]. An association between SNP (rs1143634) *IL1B* and IL-1β plasma concentration of patients undergoing various therapies has been reported [27,28]. In vitro studies have shown that this SNP influences the increase in IL-1β secretion after stimulation with lipopolysaccharide. Additionally, the occurrence of this SNP translates into an increase in the amount of the active form of IL-1β [29].

Thus far, studies have focused on the relationship of this SNP with the development of selected diseases, including cancers such as breast cancer and MM [29,30]. However, one publication presents the impact of the polymorphism studied on the cachexia risk in patients with gastric cancer (GC) [31]. Our previous study showed the impact of the TT genotype of the *KIAA1524* gene (686C>T) on shorter OS in MM patients [32].

Considering the above, this study aimed to assess the relationship between SNP of the *IL1B* gene (rs1143634) or IL-1β plasma concentration and the occurrence of nutritional disorders in patients with MM.

## 2. Materials and Methods

### 2.1. Study Group

The study group consisted of 93 consecutively admitted patients meeting the inclusion and exclusion criteria. MM patients were treated at the Department of Hematooncology and Bone Marrow Transplantation of the Medical University of Lublin from 2015 to 2020. Inclusion criteria were MM diagnosed according to SLiM CRAB criteria and International Myeloma Working Group (IMWG) recommendations, treatment naïve. The exclusion criteria were second active malignancy, autoimmunological diseases, and active infections. ISS staging criteria were used in disease staging determination. According to WHO recommendations, anemia was classified by severity into absent or mild (I°) (Hgb 10 g/dL to lower level of normal), moderate (II°) (Hgb 8–10 g/dL), severe (III°) (Hgb 6.5–7.9 g/dL), and life-threatening (IV°) (Hgb < 6.5 g/dL) [33]. Renal function was divided into 2 categories: A if creatinine was <2 mg/dL, and B if creatinine was ≥2 mg/dL [34]. Assessment of patients` nutritional status was performed according to criteria proposed by ESPEN (cachexia) and GLIM (malnutrition) before the start of treatment [13,35]. This study was designed based on the STROBE guidelines. Local ethical approval was obtained from the Bioethical Committee at the Medical University of Lublin (consent number: KE-0254/26/2015). This study was conducted in strict adherence to the principles of the Declaration of Helsinki.

### 2.2. Blood Collection

A total of 5 mL of peripheral blood was collected into EDTA-containing tubes from each patient participating in this study prior to the treatment in duplicate. The second sample was centrifuged for 15 min at 1000× *g* to obtain plasma. Samples were stored at −80 °C until laboratory analysis.

### 2.3. Genotyping

DNA was purified from 200 µL of whole blood using the column method according to the manufacturer’s protocol (DNA Blood Mini Kit, Qiagen, Hilden, Germany). Evaluation of the quality and quantity of the obtained samples was performed using the NanoDrop Lite Spectrophotometer (Thermo Fisher Scientific, Waltham, MA, USA). The genotyping analysis was based on the real-time PCR method and performed on a StepOnePlus device (Applied Biosystems, Foster City, CA, USA). According to the manufacturer’s recommendations, the Genotyping Master Mix and TaqMan probes specific for the *IL1B* SNP (rs1143634) (Thermo Fisher Scientific, Waltham, MA, USA) were used. The thermal cycling protocol was based on the manufacturer’s protocol. All sample tests were performed in triplicate. After the amplification, the obtained genotypes were analyzed with StepOne Software v2.3 (Applied Biosystems, Waltham, MA, USA). Additionally, 10% of the samples were randomly selected and reanalyzed in a sequencing device (3500 Genetic Analyzer, Life Technologies, Carlsbad, CA, USA). A total of 100% consistency of the results was obtained.

### 2.4. Assessment of IL-1β Plasma Concentration

The ELISA technique was used to assess concentration of IL-1β in plasma samples (Human IL-1β ELISA Kit, Cat. No.BMS224-2, Thermo Fisher Scientific, Waltham, MA, USA) according to the manufacturer’s recommendations. The detection range of IL-1β was 0–3.9 pg/mL and the sensitivity was 0.3 pg/mL. Measurement of the optical density (OD) at 450 nm and calculation of the standard curve 5-parameter curve equation and IL-1β concentration were carried out using a Multiskan FC Multiplate Photometer (Thermo Scientific, Waltham, MA, USA). Plasma samples that exceeded the highest concentration of IL-1β were reanalyzed at a higher sample dilution. A Wellwash Versa (Thermo Scientific, Waltham, MA, USA) automatic washer was used. 

### 2.5. Bioelectrical Impendence Analysis 

Body composition parameters, including fat-free mass (FFM), were obtained with bioelectrical impedance analysis (BIA). Measurements were performed at supine position in the morning (fasting conditions). Hydration status was controlled within BIA results (no significant deviations were noted). An ImpediMed SFB7 BioImp v1.55 device (Pinkenba, QLD, Australia) was used in BIA measurements. 

### 2.6. Statistical Analysis 

MedCalc 15.8 software (MedCalc Software, Ostend, Belgium) was used to analyze the acquired data statistically. In sample size calculation, to reject the null hypothesis, the alpha error was set to 5%, and to achieve an acceptable level of statistical power equal to 80%, the beta error was set at 20%. Calculation was conducted based on a comparison of the percentages of patients with different variants of SNP of the IL1B gene and cachexia as a primary endpoint. Considering the percentage of patients with cachexia in groups with CC genotype (47.1%) and with CT or TT genotype (9.5%), and the ratio of sample sizes in compared groups (1.21), the minimal study group was estimated as 55 patients. A chi-square test was used to assess the *IL1B* genotypes’ distribution and IL-1β plasma level depending on the selected demographic, clinical, and molecular variables. Results with a *p*-value < 0.05 were considered statistically significant. Univariable analysis of the risk of malnutrition and cachexia depending on demographic, clinical, and genetic factors was performed using the odds ratio (OR) test accompanied by OR and 95% confidence interval (95% CI) calculation. Multivariable analysis of the risk of malnutrition and cancer cachexia depending on demographic, clinical, and genetic factors was performed using logistic regression for the calculation of OR and 95% CI (results were adjusted to variables indicated by the backward elimination method in which statistically significant variables from the univariable analysis were included; for malnutrition: ISS stage; albumin; for cachexia: 17p/TP53 deletion; albumin level, IL-1 beta plasma concentration/*IL1B* genotype (CC vs. CT or TT)). Univariable analysis of OS and progression-free survival (PFS) was performed using the two-sided log-rank test (with the calculation of the hazard ratio HR and 95% CI) and visualized with Kaplan–Meier curves. Multivariable OS and PFS analysis was performed using Cox proportional hazard regression models (results were adjusted to variables indicated by the backward elimination method in which statistically significant variables from the univariable analysis were included; for OS: diagnosis; *IL1B* genotype (CC vs. CT or TT); for PFS: aHSCT; albumin level). Forest plot graphs were used to illustrate the results of the multivariable analysis. The analysis of receiver operating characteristic (ROC) curves was used to determine the cut-off points and to assess the diagnostic usefulness of IL-1β plasma concertation in differentiating the nutritional status of MM patients. 

## 3. Results

### 3.1. Characteristics of the Study Group

The study group consisted of 93 newly diagnosed and treatment-naïve MM patients (52.7% were females). The median age of patients was 66 years (range: 37–87 years). MM with a monoclonal component constituted almost 90% of the study group. ISS grade 2 or 3 was reported in 31.2% and 37.6% of cases, respectively. The median BMI was 26.59 (range: 14.33–56.82). Nearly half of the patients (49.5%) experienced significant weight loss before MM diagnosis. In the first line of chemotherapy, patients received schemes based on thalidomide—CTD (cyclophosphamide + thalidomide + dexamethasone), bortezomib—V(C)D (bortezomib + cyclophosphamide + dexamethasone), or bortezomib and thalidomide—VTD (bortezomib + thalidomide + dexamethasone) in 29%, 28%, and 43% of cases, respectively. The study group characteristics are presented in Table 1.

### 3.2. Factors Affecting the Risk of Malnutrition

In our study, malnutrition was observed in 68.82% of patients.

#### 3.2.1. Univariable Analysis 

According to the univariable analysis, significantly higher risk of malnutrition was observed in patients with stage 3 disease according to the ISS classification (OR = 4.23; *p* = 0.0089), poor performance status (PS:2–4) (OR = 188.42; *p* = 0.0003), lower levels of albumin (OR = 5.14; *p* = 0.0040), and elevated CRP (OR = 2.98; *p* = 0.0368). 

#### 3.2.2. Multivariable Analysis

**Figure 1:**
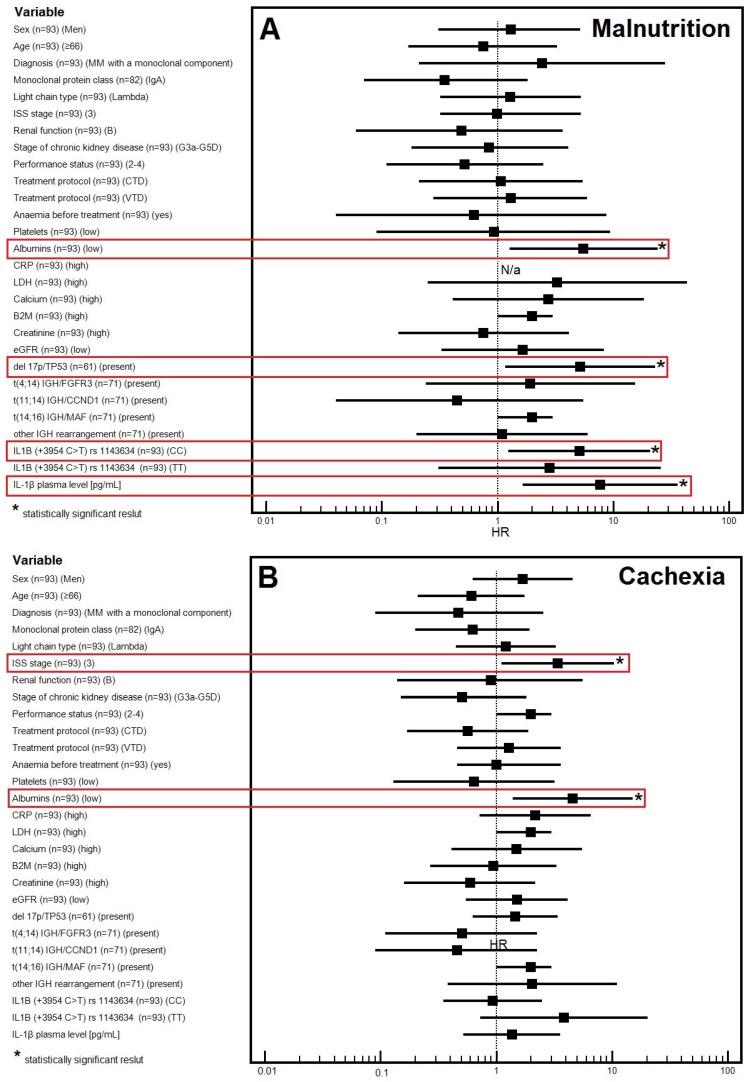
Forest plot showing the results of multivariate analysis for malnutrition (**A**) and cachexia (**B**).

**Figure 2:**
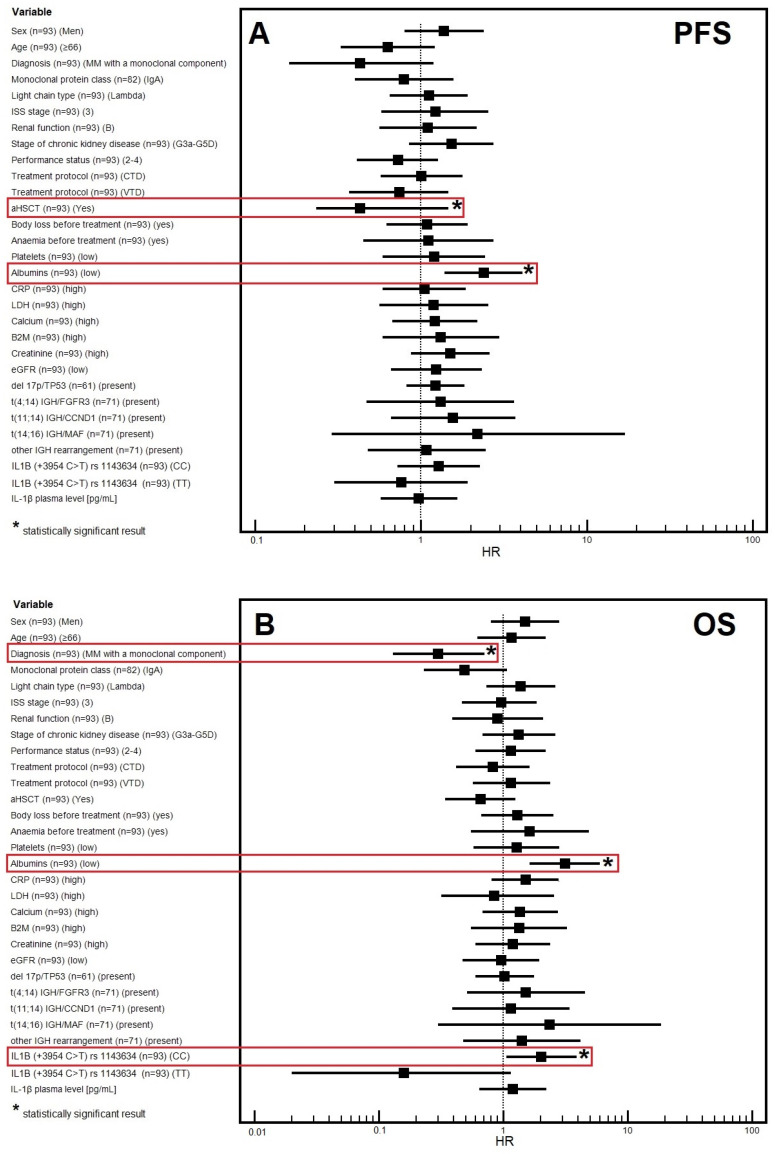
Forest plot showing the results of multivariate analysis for progression-free survival (**A**), and overall survival (**B**).

The multivariable analysis showed a significantly higher risk of malnutrition in patients with stage 3 disease according to the ISS classification (OR = 3.39) and lower albumin levels (OR = 4.56; *p* = 0.0125) (Table 2; Figure 1A and Figure 2). 

### 3.3. Factors Affecting the Risk of Cachexia 

Cachexia was noted in 30.1% of MM patients.

#### 3.3.1. Univariable Analysis 

According to the univariable analysis, significantly higher risk of cachexia was observed in patients with stage 3 disease according to the ISS classification (OR = 2.6; *p* = 0.0400), lower albumin levels (OR = 7.04; *p* = 0.0001), increased LDH (OR = 4.16; *p* = 0.0394), elevated creatinine (OR = 2.61; *p* = 0.0406), and with the presence of 17p/TP53 deletion (OR = 4.62; *p* = 0.0157). Moreover, patients with the CC genotype of the *IL1B* gene had significantly more than 8-fold higher risk of cachexia (OR = 8.44; *p* = 0.0003). Similarly, a significantly more than 8-fold higher risk of cachexia was noted in patients with higher IL-1β plasma levels (OR = 8.40; *p* = 0.0001). 

#### 3.3.2. Multivariable Analysis

The multivariable analysis showed a significantly higher risk of cachexia in patients with lower albumin levels (OR = 5.54; *p* = 0.0225), with the presence of 17p/TP53 deletion (OR = 5.20; *p* = 0.0307), with the CC genotype of *IL1B* gene (OR = 5.11; *p* = 0.0233), and with higher levels of IL-1β (OR = 7.76; *p* = 0.0092) (Table 2; Figure 1B).

### 3.4. Progression-Free Survival

#### 3.4.1. Univariable Analysis

**Figure 3:**
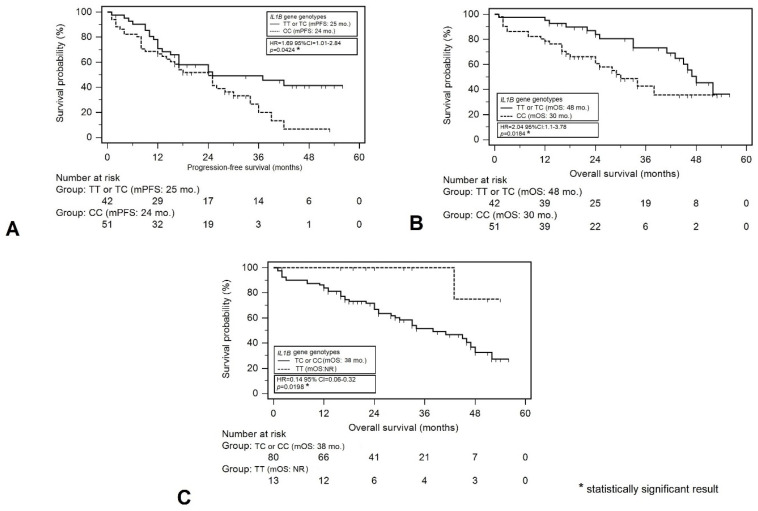
Kaplan–Meier curves presenting the relationship between *IL1B* gene genotypes and progression-free survival (**A**) or overall survival (**B**,**C**).

According to the univariable analysis, the factors significantly correlated with higher risk of PFS reduction were male sex (HR = 1.70; *p* = 0.0402), higher stage of chronic kidney disease (>G2: HR = 2.42; *p* = 0.0004), anemia before treatment (HR = 2.21; *p* = 0.0385), low albumin level (HR = 2.69; *p* = 0.0001), and high level of creatinine (HR = 2.21; *p* = 0.0017). A significantly lower risk of PFS reduction in patients treated with VTD, as compared to those treated with CTD or V(C)D schemes (HR = 0.51; *p* = 0.0387) and aHSCT (HR = 0.37; *p* = 0.0005), was found. Additionally, a significantly higher risk of PFS reduction was observed in patients with the CC variant of the *IL1B* gene (rs1143634) as compared to patients with other variants (median PFS: 24 vs. 25 months; HR = 1.69; *p* = 0.0424) (Figure 3A). 

#### 3.4.2. Multivariable Analysis

A multivariable analysis revealed a significantly lower risk of PFS reduction in patients in whom aHSCT was applied (HR = 0.43; *p* = 0.0065). On the other hand, patients with lower levels of albumin had significantly higher risk of PFS reduction (HR = 2.40; *p* = 0.0017).

Detailed data on the relationship between selected demographic, clinical, and molecular variables and PFS are presented in Table 3.

### 3.5. Overall Survival

#### 3.5.1. Univariable Analysis

The univariable analysis identified the following factors as significantly related to a higher risk of death: higher stage of chronic kidney disease (>G2: HR = 2.08; *p* = 0.0153), low albumin levels (HR = 2.68; *p* = 0.0007), and high creatinine levels (HR = 2.04; *p* = 0.0182). On the other hand, a significantly lower risk of death was observed in the subjects with MM with monoclonal component (HR = 0.33; *p* = 0.0011). Moreover, we observed a higher risk of death in patients with the CC variant of the *IL1B* gene (rs1143634) as compared to those with other genotypes (median OS: 30 months vs. 48 months; HR = 2.04; *p* = 0.0184) (Figure 3B). On the other hand, a significantly lower risk of death was observed in the subjects with the TT variant of the *IL1B* gene (rs1143634) as compared to carriers of the C allele (HR = 0.14; *p* = 0.0198) (Figure 3C).

#### 3.5.2. Multivariable Analysis

The multivariable analysis confirmed the independent prognostic value of the following factors: MM with a monoclonal component (HR = 0.30; *p* = 0.076), low albumin levels (HR = 3.14; *p* = 0.0006), and CC genotype of *IL1B* gene (rs1143634) (HR = 2.03; *p* = 0.0337). A graphical representation of the results of the multivariable analysis of PFS and OS is shown in Figure 2A,B.

Detailed data on the relationship between selected demographic, clinical, and molecular variables and OS are presented in Table 3.

### 3.6. The Association between Gender and Demographic, Clinical, and Molecular Factors

We observed no statistically significant differences in IL-1β concentration and IL1B SNP depending on gender and other demographic, clinical, and molecular variables (Table S1). 

### 3.7. The Association between IL1B Genotypes’ Distribution and Demographic, Clinical, and Molecular Factors

**Figure 4:**
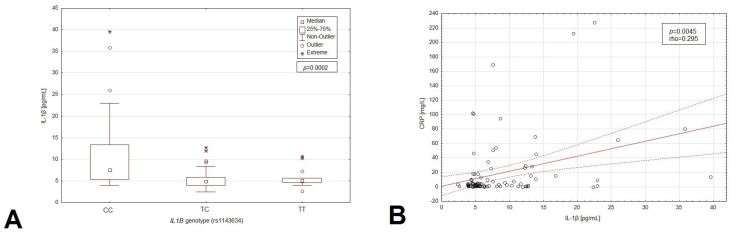
Box–whisker graph presenting a comparison of concertation of the IL-1β depending on genotypes of the SNP of *IL1B* gene (**A**). Distribution plot presenting the correlation between IL-1β and CRP plasma concentrations (**B**).

Elevated levels of CRP were noted to be significantly more common in patients with the CC genotype than in the carriers of the T allele (54.9% vs. 29.2%; *p* = 0.0003). We observed significantly higher IL-1β concentrations in the carriers of CC compared to the other variants of the *IL1B* gene (68.6% vs. 50.7%; *p* = 0.0002; Figure 4). The detailed data are presented in the Table S2.

Patients with the CC genotype had significantly higher IL-1β concentrations in plasma (7.56 vs. 4.97 pg/mL; *p* < 0.0001). Moreover, patients with CC genotype had significantly higher CRP concentration as compared to those with other variants of the *IL1B* gene (8.3 vs. 2 mg/L; *p* = 0.0014). Significantly lower albumin level was noted in CC genotype carriers (3.60 vs. 3.85 g/dL; *p* = 0.0165) (Table S3). 

Patients with elevated CRP had significantly higher IL-1β plasma concentration than patients with normal CRP levels (8.35 vs. 5.38 pg/mL; *p* = 0.0004) (Table S4). Moreover, we found a significant, weak, positive correlation between CRP and IL-1β plasma concentration (r = 0.295; *p* = 0.0045; Table S5; Figure 4B).

### 3.8. Diagnostic Usefulness of the Assessment of the IL1B SNP (rs1143634) and IL-1β Concentration in Predicting Nutritional Disorders 

**Figure 5:**
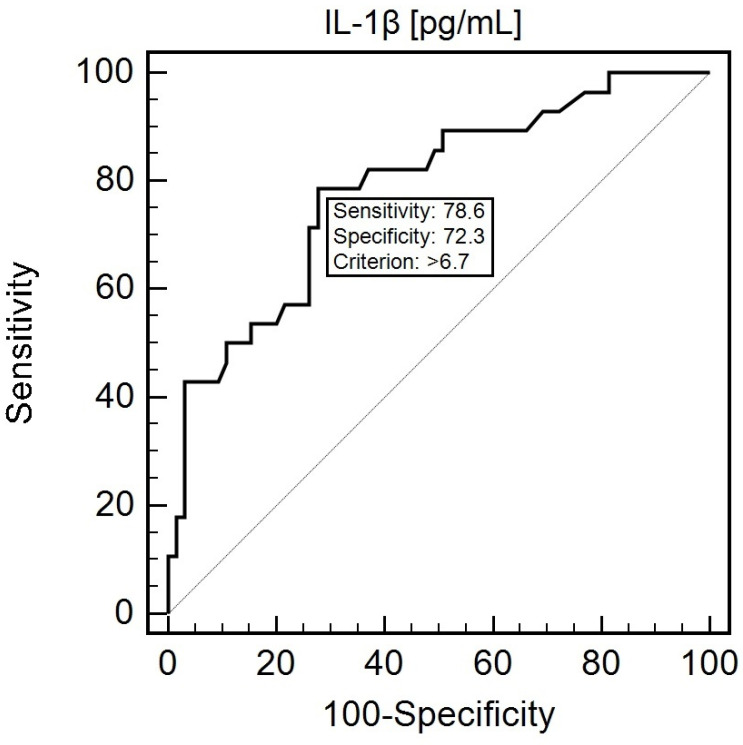
ROC curve presenting the assessment of the diagnostic usefulness of plasma IL-1β concentration in predicting the occurrence of cachexia. Abbreviations: IL-1β—interleukin 1 beta.

The assessment of *IL1B* SNP and IL-1β plasma concentration demonstrated a significant diagnostic usefulness in the prediction of cachexia. The CC genotype of the *IL1B* gene was characterized with 85.7% sensitivity and 58.5% specificity in the prediction of cachexia (AUC = 0.72; *p* < 0.0001). At the cut-off of >6.7 pg/mL, 78.6% sensitivity and 72.3% specificity in the prediction of cachexia was noted (AUC = 0.788; *p* ≤ 0.0001; Figure 5). Joint analysis of IL-1β and CRP levels was characterized by high diagnostic usefulness in predicting the risk of cancer cachexia (AUC = 0.956; *p* < 0.0001).

The assessment of *IL1B* SNP and IL-1β plasma concentration demonstrated a nonsignificant diagnostic usefulness in the prediction of malnutrition. The CC genotype of *IL1B* gene was characterized with 56.2% sensitivity and 48.2% specificity in the prediction of malnutrition (AUC = 0.52; *p* = 0.6894). At the cut-off of >5.1 pg/mL, 75% sensitivity and 48.3% specificity in the prediction of malnutrition was observed (AUC = 0.59; *p* = 0.1634). 

## 4. Discussion

An important phenomenon accompanying cachexia is the inflammatory process, during which the secretion of proinflammatory cytokines is observed, including IL-1β, Il-6, IL8, TNF-α, and interferon-gamma (INF-γ) [33]. Moreover, in the BM of MM patients, cancer cells stimulate the secretion of IL-1β and IL-6 [21]. To the best of our knowledge, this study is the first to describe this type of relationship in MM. Due to the lack of this type of papers in MM, we decided to compare our results with those obtained in GC, gastroesophageal junction (GEJ) cancer, or chronic obstructive pulmonary disease (COPD). As in the case of nutritional disorders, there are no studies in the available literature describing the association of the SNP we examined with OS in patients with MM.

In our study including de novo diagnosed and previously untreated MM patients, cachexia was noted in 30.1% and malnutrition was observed in 68.8% of cases. Mallard et al. conducted a study on 31 patients with MM, 30 with Hodgkin’s lymphoma, and 84 with non-Hodgkin’s lymphoma. They described that cachexia was noted in 38% of patients treated due to hematological malignancies [36]. In turn, in the study conducted by Olleros et al. in a group of 58 patients with MM, 45.4% were moderately or severely malnourished [10]. Kim et al. conducted a study on 216 patients with MM. Using the Subjective Global Assessment (SGA) scale, they showed that 71% of patients had malnutrition, of which 23% had severe malnutrition requiring nutritional intervention [8]. Meanwhile, Garzón Herazo et al. conducted a study on 124 patients with MM treated with aHSCT. Based on the nutritional risk index (NRI < 97.5) calculated before aHSCT, they found that 35% of patients were moderately or severely malnourished Thus, our data are consistent with the available literature.

The presence of rapidly proliferating cancer cells contributes to the development of a systemic inflammatory process that underlies the processes related to the remodeling of muscle and fat tissue [37]. IL-1β promotes muscle fiber breakdown by activating the nuclear factor kappa B (NF-κB) pathway and increasing IL-6 levels. TNF-α, also called cachectin, IL-6, IL-1β, and IFN-γ, activates the NF-κB pathway and promotes the transcription of E3 ubiquitin-proteasome ligase in muscle atrophy [23]. Moreover, in the course of cachexia, browning of adipose tissue is observed, which is mainly mediated by proinflammatory cytokines, e.g., TNF-α, IL-6, and IL-1β [38,39]. Furthermore, IL-1β plays an important role in activating lipolysis by promoting adipose tissue triglyceride lipase (ATGL) and hormone-sensitive lipase (HSL). High levels of IL-1β promote the production of lipid mobilizing factor (LMF), which stimulates increased lipolysis [23]. Gherardi et al. showed that an increased concentration of IL-1β is observed in the serum of MM patients [40,41]. In our study, an increase in IL-1β levels in the plasma of MM patients was associated with an increase in the risk of developing cachexia. Bębnowska et al. showed that IL-1β stimulates the production of IL-6 and IL-10 in patients with MM [42]. Kline et al., in studies using cultures of stromal cells derived from the bone marrow of MM patients, showed that in addition to IL-6, IL-1β also induces an increase in the secretion of IL-8 (to the greatest extent), monocyte chemoattractant protein (MCP-1), and granulocyte-macrophage colony-stimulating factor (GM-CSF) [43].

The available literature focuses on the association of SNPs of genes encoding proinflammatory cytokines, i.e., *IL-6*: rs1800795 (-174G>C), *TNF-α*: rs1800629 (-308G>A), or *IL1B* rs16944 (-511C>T), with the development of MM [44,45,46]. Similarly, in the case of the rs1143634, SNP we studied, available studies describe its relationship with the risk of developing selected cancers, including MM [28,29]. It should be emphasized that in the literature, there are no studies describing the association of SNPs of genes encoding proinflammatory cytokines, including those located within *IL1B*, in particular rs1143634, with the risk of nutritional disorders in MM [47,48]. Zhang et al. performed an *IL1B* SNP analysis (rs1143634) on a group of 214 patients with locally advanced gastric cancer. The T allele of this gene was significantly more common in patients with cachexia compared to patients without cachexia (12.1% vs. 5.7%). Therefore, the presence of the T allele was associated with a significant, over 2.5-fold higher, risk of cachexia (OR = 2.51; 95% CI: 1.18–5.35) [31]. In another study by Jatoi et al., 44 patients with confirmed gastric cancer and gastroesophageal junction cancer with metastases were enrolled, and the *IL1B* SNP (rs1143634) was analyzed. Compared to patients with the CC genotype, carriers of the T allele showed a significant increase in body weight during the study and a significantly lower risk of death (HR = 0.3) [49]. In the study conducted by Broekhuizen et al., SNP rs16944 *IL1B* (-511T>C) was analyzed in a group of 99 patients with COPD. The occurrence of the CC genotype was associated with a higher incidence of cachexia compared to other genotype variants (46.3% vs. 22.7%) [50]. Therefore, our results, in which MM patients with the CC genotype had a higher risk of cachexia and shorter OS compared to carriers of the T allele, are consistent with those obtained by Jatoi et al. and Broekhuizen et al., while contrary to those presented in the work of Zhang et al. (only regarding risk of cachexia). However, it should be noted that the study conducted by Zhang et al. involved Asian patients, in whom the allele distribution was substantially different from that observed in our study (the TT genotype was not noted in any patient, while in our study it was recorded in 13 patients). Additionally, the study mentioned above involved GC patients, whereas ours included MM patients; therefore, the studied SNP could have different clinical consequences (including risk of cachexia). The importance of the above differences (race, cancer type) has already been indicated in the literature [51,52].

As in the case of nutritional disorders, there are no studies in the available literature describing the association of the SNP we examined with OS in patients with MM. Therefore, we report the association of other SNPs located in the gene we studied with the survival of MM patients. In a study conducted by Vangsted et al. in 348 MM patients, SNP rs1143627 of the *IL1B* gene (-31C>T) was analyzed. In this group, 185 patients were treated with high doses of melphalan and aHSCT, while 163 patients were treated with other regimens. The presence of the C allele was associated with a significantly longer OS (mOS: 80.1 vs. 48.5 months; HR = 0.6; 95% CI: 0.5–0.9) and a nonsignificantly longer time to treatment failure (TTF) compared to the presence of the TT genotype (29.9 vs. 27.2 months; HR = 0.9; 95% CI: 0.6–1.2) [53]. On the other hand, in our study, the CC genotype was associated with shorter OS compared with other variants of *IL1B* (rs1143634) genotypes. Although we examined a different SNP, localization in the same gene suggests its involvement in cancer progression and, therefore, the potential usefulness of determining this gene’s status in prognostication.

In another study, Vangsted et al. analyzed the association of several SNPs of the *IL1B* gene, including rs16944 (-511C>T), rs4848306 (-3737 C>T), and rs1143623 (-1464 G>C) with TTF and OS in patients with MM. The study included 348 patients with MM treated with high doses of melphalan and stem cells, of which 146 patients received interferon-alpha (INF-α) maintenance treatment. The disease recurred in 243 patients, and among this group, 177 people were treated with thalidomide and 74 received bortezomide. In the case of the SNP rs4848306 of the *IL1B* gene, in T allele carriers compared to CC homozygotes, a significantly higher risk of death (62.1 vs. 85.8 months; HR = 1.8; 95% CI: 1.2–2.6) and a significantly higher risk of TTF shortening (26 vs. 32.8 months; HR = 1.4; 95% CI: 1–1.9) was observed. In turn, in the case of the SNP rs16944 of the *IL1B* gene, in carriers of the TT homozygotes compared to CC or CT genotypes, a significantly lower risk of death was observed (93.9 vs. 54.9 or 74.4 months; HR = 0.5; 95% CI: 0.3–1) and a nonsignificant difference in the risk of shortening TTF (34.4 vs. 27.8 or 27.6 months; HR = 1; 95% CI: 0.6–1.5). However, in the case of rs1143623 of the *IL1B* gene, a significantly lower risk of death was observed in carriers of the C allele compared to GG homozygotes (81.6 vs. 56.7 months; HR = 0.6; 95% CI: 0.5–0.9) and a nonsignificant difference in the risk of TTF shortening (28.9 vs. 28.4 months; HR = 1; 95% CI: 0.8–1.3) [46]. 

Also, in this case, we examined different SNP; however, considering the explanation mentioned in the previous paragraph, utilization of determining this gene’s status as a prognostic factor could be suggested. In our previous study including 128 patients with MM diagnosed de novo, we noted a higher risk of death in the carriers of the TT genotype (rs2278911; 686C>T) of the *KIAA1524* gene (mOS: 8 months vs. 45 months; HR = 0.53). Moreover, patients with the CC genotype had a significantly lower risk of OS reduction compared to other *KIAA1524* genotypes (HR = 0.41). In silico analysis showed that the *KIAA1524* gene correlates significantly with IL-6, one of the key proinflammatory cytokines, which is further evidence of the link between severe inflammation and unfavorable prognosis in MM [33].

It should be considered that performing SNP analysis may have an advantage over simply measuring CRP or IL-1β levels because its determination is independent of the patient’s current status (presence of inflammation unrelated to cancer and/or the development of other diseases, nutrition, comorbidities, previous treatment affecting the immune system, etc.). For this reason, as a predisposition factor to the development of cachexia or a worse prognosis, SNPs are not susceptible to confounding factors. At the same time, in the era of technological progress, the determination of SNPs is relatively simple and affordable. On the other hand, the fact that the indicated SNPs do not reflect the current state of the immune system, but, rather, some predispositions, may be perceived as a disadvantage.

A limitation of the study includes the lack of determination of other proinflammatory cytokines that are involved in the development of nutritional disorders. To date, numerous SNPs were associated with the development of cachexia or poor survival in various cancers; thus, combined with our results, this may suggest that the studied SNP may also be a useful predictive (regarding nutritional disorders) and prognostic factor in patients with MM. 

In the context of OS, conclusions regarding the advantage of IL1B SNP over IL-1β concentration (categorized for this analysis) determination can be drawn based on the comparison of HR values. Higher HR values (and, above all, statistically significant) were obtained for SNP. 

Potential practical implications of the conducted research include the utilization of IL-1β concentration or *IL1B* SNP (rs1143634) determination in the assessment of the predispositions to nutritional deficiencies, facilitation of the diagnosis of such type of disorders, the development of anti-IL1B therapy to treat or prevent malnutrition and cachexia, assessment of patients regarding the level of IL-1β or *IL1B* SNP (rs1143634) to assess predispositions, diagnosis of this type of disorder, implementation of appropriate supportive treatment (e.g., nutritional) in diagnosed or at-risk patients, the development of anti-IL1B therapy to treat or prevent nutritional deficiencies, and, finally, for prognostic purposes in MM patients. Moreover, determining the level of IL-1β with other parameters increases the diagnostic utility in assessing the risk of developing cachexia.

## 5. Conclusions

Determination of the *IL1B* SNP (rs1143634) and plasma concentration of IL-1β may be useful in the assessment of the risk of cachexia and prognosis in patients with MM. 

## Figures and Tables

**Table 1:** General characteristics of the study group.

Factor		Study Group (n = 93)
Gender	Male	44 (47.3%)
	Female	49 (52.7%)
Age (years)	Mean ± standard deviation, median (range)	64.3 ± 9.83 66 (37–87)
	≥65	49 (52.7%)
	<65	44 (47.3%)
Myeloma type	IgG	55 (59.1%)
	IgA	27 (29%)
	Light chains	11 (11.8%)
Light chain type	Kappa	57 (61.3%)
	Lambda	36 (38.7%)
ISS stage	1	29 (31.2%)
	2	29 (31.2%)
	3	35 (37.6%)
Renal function	A	80 (86%)
	B	13 (14%)
Performance status	0	7 (7.5%)
	1	37 (39.8%)
	2	37 (39.8%)
	3	10 (10.8%)
	4	2 (2.2%)
BMI (kg/m^2^)	Mean ± standard deviation, median (range)	27.06 ± 5.98 26.59 (14.53–56.82)
Body weight loss	Yes	46 (49.5%)
	No	47 (50.5%)
	5%	14 (30.4%)
	10%	32 (69.6%)
Body weight loss (kg)	Mean ± standard deviation, median (range)	5.6 ± 2.4 5 (0–17)
Anemia grade before treatment (WHO)	Absent or I°	51 (54.8%)
	II°	27 (29%)
	III°	14 (15.1%)
	IV°	1 (1.1%)
Treatment protocol	CTD	27 (29%)
	V(C)D	26 (28%)
	VTD	40 (43%)
aHSCT	No	55 (59.1%)
	Yes	38 (40.9%)
del 17p/TP53 *No data: n = 32*	Absent	45 (73.8%)
	Present	16 (26.2%)
t(4;14) IGH/FGFR3 *No data: n = 22*	Absent	61 (85.9%)
	Present	10 (14.1%)
t(11;14) IGH/CCND1 *No data: n = 22*	Absent	63 (88.7%)
	Present	8 (11.3%)
t(14;16) IGH/MAF *No data: n = 31*	Absent	61 (98.4%)
	Present	1 (1.6%)
Other *IGH* rearrangement *No data: n = 22*	Absent	60 (84.5%)
	Present	11 (15.5%)

Abbreviations: aHSCT—autologous hematopoietic stem cell transplantation; BMI—body mass index; CTD—cyclophosphamide, thalidomide, dexamethasone; IGH—immunoglobulin heavy chain; ISS—Multiple Myeloma International Staging System; WHO—World Health Organization; V(C)D—bortezomib, (cyclophosphamide), dexamethasone; VTD—bortezomib, thalidomide, dexamethasone.

**Table 2:** Malnutrition and cancer cachexia occurrence depending on selected demographic, clinical, and molecular variables.

Variable	Malnutrition				Cancer Cachexia			
			Univariable	Multivariable			Univariable	Multivariable
	No (n = 29)	Yes (n = 64)	OR [95% CI] *p*	OR [95% CI] *p*	No (n = 65)	Yes (n = 28)	OR [95% CI] *p*	OR [95% CI] *p*
Gender								
Men	11 (25%)	33 (75%)	1.74 [0.71–4.27]	1.70 [0.63–4.58]	28 (63.6%)	16 (36.4%)	1.76 [0.72–4.31]	1.31 [0.31–5.21]
Women	18 (36.7%)	31 (63.3%)	0.2248	0.2891	37 (75.5%)	12 (24.5%)	0.2149	0.7170
Age								
≥65	14 (28.6%)	35 (71.4%)	1.29 [0.54–3.11]	0.61 [0.21–1.76]	31 (63.3%)	18 (36.7%)	1.97 [0.79–4.92]	0.76 [0.17–3.28]
<65	15 (34.1%)	29 (65.9%)	0.5666	0.3586	34 (77.3%)	10 (22.7%)	0.1445	0.7101
Diagnosis								
MM with a monoclonal component	27 (32.9%)	55 (67.1%)	0.45 [0.09–2.24]	0.47 [0.09–2.57]	57 (69.55)	25 (30.5%)	1.17 [0.29–4.78]	2.43 [0.21–28.07]
Light chain disease	2 (18.2%)	9 (81.8%)	0.3316	0.3856	8 (72.7%)	3 (27.3%)	0.8273	0.4757
Monoclonal protein class								
IgA	8 (29.6%)	19 (70.4%)	0.80 [0.29–2.16]	0.63 [0.2–1.94]	17 (63%)	10 (37%)	0.64 [0.24–1.70]	0.35 [0.07–1.82]
IgG	19 (34.5%)	36 (65.5%)	0.6565	0.4192	40 (72.7%)	15 (27.3%)	0.3684	0.2138
*N/a: n = 11*								
Light chain type								
Lambda	10 (27.8%)	26 (72.2%)	1.30 [0.52–3.24]	1.21 [0.45–3.28]	23 (63.9%)	13 (36.1%)	1.58 [0.64–3.89]	1.29 [0.32–5.28]
Kappa	19 (33.3%)	38 (66.7%)	0.5736	0.7047	42 (73.7%)	15 (26.3%)	0.3175	0.7206
ISS stage								
3	5 (14.3%)	30 (85.7%)	4.23 [1.43–12.49]	3.39 [1.11–10.41]	20 (57.1%)	15 (42.9%)	2.60 [1.04–6.45]	0.99 [0.22–4.45]
1, 2	24 (41.4%)	34 (58.6%)	0.0089 *	0.0327 *	45 (77.6%)	13 (22.4%)	0.0400 *	0.9880
Renal function								
B	2 (15.4%)	11 (54.6%)	2.80 [0.58–13.55]	0.90 [0.14–5.57]	7 (53.8%)	6 (46.2%)	2.56 [0.68–7.47]	0.49 [0.06–3.66]
A	27 (33.7%)	53 (66.2%)	0.2002	0.9067	58 (72.5%)	22 (27.5%)	0.1815	0.4857
Stage of chronic kidney disease								
G3a,G3b, G4, G5D	8 (24.2%)	25 (75.8%)	1.68 [0.64–4.38]	0.51 [0.15–1.82]	20 (60.6%)	13 (39.4%)	1.95 [0.78–4.85]	0.85 [0.18–4.12]
G1,G2	21 (35%)	39 (65%)	0.2864	0.3017	45 (75%)	15 (25%)	0.1506	0.8456
Performance status								
2–4	0 (0%)	49 (100%)	188.42 [10.87–3266.72]	-[-]	30 (61.2%)	19 (38.8%)	2.46 [0.97–6.24]	0.52 [0.11–2.48]
0, 1	29 (65.9%)	15 (34.1%)	0.0003 *	0.9939	35 (79.5%)	9 (20.5%)	0.0578	0.4152
Treatment protocol (1)								
CTD	5 (18.5%)	22 (81.5%)	2.51 [0.84–7.50]	0.57 [0.17–1.89]	18 (66.7%)	9 (33.3%)	1.24 [0.47–3.23]	1.07 [0.21–5.46]
V(C)D, VTD	24 (36.4%)	42 (63.6%)	0.0982	0.3625	47 (71.2%)	19 (28.8%)	0.6647	0.9381
Treatment protocol (2)								
VTD	10 (35.7%)	18 (64.3%)	0.74 [0.29–1.90]	1.29 [0.46–3.64]	20 (71.4%)	8 (28.6%)	0.90 [0.34–2.38]	1.30 [0.28–5.98]
CTD, V(C)D	19 (29.2%)	46 (70.8%)	0.5365	0.6288	45 (69.2%)	20 (30.8%)	0.8322	0.7354
Anemia before treatment (WHO)								
Yes	21 (28%)	54 (72%)	2.06 [0.71–5.92]	1.01 [0.28–3.61]	49 (65.3%)	26 (34.7%)	4.24 [0.90–19.90]	0.63 [0.04–8.71]
No	8 (44.4%)	10 (55.6%)	0.1813	0.9923	16 (88.9%)	2 (11.1%)	0.0666	0.7317
Platelets								
Low	3 (25%)	9 (75%)	1.42 [0.35–5.68]	0.64 [0.13–3.18]	7 (58.3%)	5 (41.7%)	1.80 [0.52–6.26]	0.93 [0.09–9.46]
Normal	26 (32.1%)	55 (67.9%)	0.6216	0.5885	58 (71.6%)	23 (28.4%)	0.3543	0.9547
Albumins								
Low	4 (11.8%)	30 (88.2%)	5.14 [1.72–17.66]	4.56 [1.39–15.03]	15 (44.1%)	19 (55.9%)	7.04 [2.64–18.76]	5.54 [1.27–24.1]
Normal	25 (42.4%)	34 (57.6%)	0.0040 *	0.0125 *	50 (84.7%)	9 (15.3%)	0.0001 *	0.0225 *
CRP								
High	6 (17.6%)	28 (82.4%)	2.98 [1.07–8.31]	2.17 [0.72–6.59]	6 (17.6%)	28 (82.4%)	N/a	N/a
Normal	23 (39%)	36 (61%)	0.0368 *	0.1705	59 (100%)	0 (0%)		
LDH								
High	0 (0%)	10 (100%)	11.37 [0.64–200.93]	-[-]	4 (40%)	6 (60%)	4.16 [1.07–16.14]	3.28 [0.25–43.74]
Normal	29 (34.9%)	54 (65.1%)	0.0972	0.9937	61 (73.5%)	22 (26.5%)	0.0394 *	0.3678
Calcium								
High	4 (19%)	17 (81%)	2.26 [0.68–7.44]	1.5 [0.41–5.51]	11 (52.4%)	10 (47.6%)	2.72 [0.99–7.48]	2.75 [0.41–18.33]
Normal	25 (34.7%)	47 (65.3%)	0.1800	0.5400	54 (75%)	18 (25%)	0.0513	0.2944
B2M								
High	23 (29.5%)	55 (70.5%)	1.59 [0.51–4.99]	0.95 [0.27–3.32]	52 (66.7%)	26 (33.3%)	3.25 [0.68–15.49]	-[-]
Normal	6 (40%)	9 (60%)	0.4234	0.9332	13 (86.7%)	2 (13.3%)	0.1390	0.9946
Creatinine								
High	7 (21.9%)	25 (78.1%)	2.01 [0.75–5.41]	0.6 [0.16–2.17]	18 (56.2%)	14 (43.7%)	2.61 [1.04–6.54]	0.76 [0.14–4.16]
Normal	22 (36.1%)	39 (63.9%)	0.1645	0.4355	47 (77%)	14 (23%)	0.0406 *	0.7545
eGFR								
Low	15 (24.6%)	46 (75.4%)	2.38 [0.96–5.92]	1.51 [0.55–4.13]	41 (67.2%)	20 (32.8%)	1.46 [0.56–3.83]	1.65 [0.33–8.28]
Normal	14 (43.7%)	18 (56.2%)	0.0611	0.4221	24 (75%)	8 (25%)	0.4380	0.5425
del 17p/TP53								
Present	2 (12.5%)	14 (87.5%)	4.25 [0.86–21.04]	1.47 [0.63–3.41]	8 (50%)	8 (50%)	4.62 [1.33–16.02]	5.2 [1.17–23.21]
Absent	17 (37.8%)	28 (62.2%)	0.0762	0.3678	37 (82.2%)	89 (17.8%)	0.0157 *	0.0307 *
*No data: n = 32*								
t(4;14) IGH/FGFR3								
Present	4 (40%)	6 (60%)	0.63 [0.16–2.50]	0.51 [0.11–2.27]	7 (70%)	3 (30%)	1.11 [0.26–4.79]	1.92 [0.24–15.49]
Absent	18 (29.5%)	43 (70.5%)	0.5085	0.3736	44 (72.1%)	17 (27.9%)	0.8896	0.5393
*No data: n = 22*								
t(11;14) IGH/CCND1								
Present	4 (50%)	4 (50%)	0.40 [0.09–1.77]	0.46 [0.09–2.27]	6 (75%)	2 (25%)	0.83 [0.15–4.52]	0.45 [0.04–5.54]
Absent	18 (28.6%)	45 (71.4%)	0.2280	0.3404	45 (71.4%)	18 (28.6%)	0.8326	0.5366
*No data: n = 22*								
t(14;16) IGH/MAF								
Present	1 (100%)	0 (0%)	0.14 [0.005–3.70]	-[-]	1 (100%)	0 (0%)	0.82 [0.03–20.99]	-[-]
Absent	21 (30%)	49 (70%)	0.2424	0.9920	50 (71.4%)	20 (28.6%)	0.9052	0.9950
*No data: n = 22*								
Other IGH rearrangement								
Present	2 (18.2%)	9 (81.8%)	2.25 [0.44–11.41]	2.05 [0.38–11.03]	7 (63.6%)	4 (36.4%)	1.57 [0.40–6.09]	1.1 [0.2–6.01]
Absent	20 (33.3%)	40 (66.7%)	0.3276	0.4008	44 (73.3%)	16 (26.7%)	0.5133	0.9094
*No data: n = 22*								
*IL1B* genotype (rs1143634)								
CC	15 (29.4%)	36 (70.6%)	1.20 [0.50–2.89]	0.93 [0.35–2.48]	27 (52.9%)	24 (47.1%)	8.44 [2.63–27.15]	5.11 [1.25–20.92]
TT or TC	14 (33.3%)	28 (66.7%)	0.6847	0.8935	38 (90.5%)	4 (9.5%)	0.0003 *	0.0233 *
*IL1B* genotype (rs1143634)								
TT	2 (15.4%)	11 (84.6%)	2.80 [0.58–13.55]	3.87 [0.73–20.43]	11 (84.6%)	2 (15.4%)	0.38 [0.08–1.83]	2.81 [0.31–25.77]
TC or CC	27 (33.7%)	53 (66.2%)	0.2002	0.1110	54 (67.5%)	26 (32.5%)	0.2263	0.3607
IL-1β plasma level [pg/mL]								
Low	17 (36.2%)	30 (63.8%)	0.62 [0.256–1.51]	1.37 [0.52–3.59]	42 (89.4%)	5 (10.6%)	8.40 [2.82–25.05]	7.76 [1.66–36.31]
High	12 (26.1%)	34 (73.9%)	0.2956	0.5229	23 (50%)	23 (50%)	0.0001 *	0.0092 *

*—Statistically significant result. Abbreviations: B2M—beta-2-microglobulin; CI—confidence interval; CRP—C-reactive protein; CTD—cyclophosphamide, thalidomide, dexamethasone; eGFR—estimated glomerular filtration rate; IgA—immunoglobulin A; IgG—immunoglobulin G; IGH—immunoglobulin heavy chain; IL-1β—interleukin 1 beta; ISS—Multiple Myeloma International Staging System; LDH—lactate dehydrogenase; N/a—not applicable; OR—odds ratio; WHO—World Health Organization; V(C)D—bortezomib, (cyclophosphamide), dexamethasone; VTD—bortezomib, thalidomide, dexamethasone.

**Table 3:** Survival of MM patients depending on selected demographic, clinical, and molecular variables.

Variable	Progression Free Survival			Overall Survival		
	Univariable		Multivariable	Univariable		Multivariable
	mPFS (Months) 18	HR (95% CI) *p*	HR (95% CI) *p*	mOS (Months) 25	HR (95% CI) *p*	HR (95% CI) *p*
Gender						
Men	24	1.7 (1.01–2.87)	1.38 (0.8–2.39)	34	1.50 (0.81–2.77)	1.5 (0.8–2.81)
Women	25	0.0402 *	0.2485	47	0.1911	0.2125
Age						
≥65	17	1.49 (0.89–2.50)	0.63 (0.33–1.21)	38	1.30 (0.71–2.41)	1.17 (0.62–2.2)
<65	30	0.1287	0.1687	45	0.3971	0.6286
Diagnosis						
MM with a monoclonal component	25	0.64 (0.2–1.48)	0.43 (0.16–1.19)	47	0.33 (0.12–0.93)	0.30 (0.13–0.72)
Light chain disease	15	0.2082	0.1056	16	0.0011 *	0.0076 *
Monoclonal protein class						
IgA	24	1.10 (0.59–2.02)	0.79 (0.40–1.58)	47	0.62 (0.29–1.33)	0.49 (0.23–1.07)
IgG	25	0.7522	0.5162	45	0.1780	0.0766
*N/a: n = 11*						
Light chain type						
Lambda	17	1.10 (0.64–1.90)	1.12 (0.65–1.92)	33	1.57 (0.82–3)	1.38 (0.73–2.62)
Kappa	26	0.7174	0.6845	48	0.1446	0.3214
ISS stage						
3	17	1.39 (0.8–2.40)	1.22 (0.58–2.56)	33	1.3 (0.68–2.46)	0.96 (0.8–1.86)
1, 2	26	0.2114	0.6018	45	0.4061	0.9156
Renal function						
B	15	1.75 (0.79–3.89)	1.1 (0.56–2.17)	24	1.82 (0.74–4.48)	0.9 (0.39–2.09)
A	25	0.0854	0.7773	45	0.1018	0.8109
Stage of chronic kidney disease						
G3a, G3b,G4,G5D	13	2.42 (1.36–4.31)	1.53 (0.85–2.74)	25	2.08 (1.09–3.98)	1.33 (0.68–2.62)
G1, G2	34	0.0004 *	0.1568	47	0.0153 *	0.4091
Performance status						
2–4	25	1.25 (0.75–2.11)	0.73 (0.41–1.27)	33	1.7 (0.92–3.15)	1.15 (0.6–2.21)
0, 1	24	0.3872	0.2653	47	0.0849	0.6632
Treatment protocol (1)						
CTD	24	1.68 (0.67–2.04)	1.01 (0.57–1.79)	33	0.96 (0.51–1.8)	0.83 (0.42–1.63)
V(C)D, VTD	25	0.5624	0.9609	43	0.8874	0.5900
Treatment protocol (2)						
VTD	36	0.51 (0.29–0.90)	0.74 (0.37–1.47)	38	1.03 (0.51–2.07)	1.16 (0.57–2.38)
CTD, V(C)D	17	0.0387 *	0.3923	47	0.9232	0.6752
aHSCT						
Yes	42	0.37 (0.22–0.62)	0.43 (0.24–0.79)	45	0.60 (0.32–1.1)	0.66 (0.34–1.26)
No	15	0.0005 *	0.0065 *	38	0.1094	0.2111
Body weight loss before treatment						
Yes	18	1.46 (0.87–2.45)	1.09 (0.62–1.91)	33	1.36 (0.73–2.52)	1.3 (0.67–2.53)
No	34	0.1471	0.7602	46	0.3181	0.4368
Anemia before treatment (WHO)						
Yes	18	2.21 (1.2–4.07)	1.11 (0.45–2.75)	38	1.84 (0.92–3.65)	1.64 (0.55–4.88)
No	-	0.0385 *	0.8137	48	0.1263	0.3719
Platelets						
Low	12	1.82 (0.78–4.24)	1.2 (0.59–2.43)	24	1.60 (0.65–3.94)	1.28 (0.58–2.83)
Normal	25	0.0753	0.6086	43	0.2253	0.5421
Albumins						
Low	12	2.69 (1.49–4.84)	2.4 (1.39–4.14)	28	2.68 (1.33–5.37)	3.14 (1.64–6.02)
Normal	37	0.0001 *	0.0017 *	48	0.0007 *	0.0006*
CRP						
High	24	1.48 (0.85–2.58)	1.05 (0.59–1.86)	30	1.79 (0.92–3.49)	1.51 (0.81–2.84)
Normal	26	0.1268	0.8611	46	0.0560	0.1973
LDH						
High	12	1.71 (0.68–4.29)	1.19 (0.56–2.54)	22	1.55 (0.51–4.74)	0.85 (0.32–2.56)
Normal	25	0.1468	0.6489	45	0.3468	0.7491
Calcium						
High	25	1.24 (0.66–2.31)	1.21 (0.67–2.19)	25	1.62 (0.46–3.45)	1.37 (0.68–2.77)
Normal	25	0.4689	0.5284	46	0.1541	0.3748
B2M						
High	24	1.85 (0.97–3.52)	1.32 (0.59–2.96)	38	1.76 (0.85–3.64)	1.34 (0.55–3.27)
Normal	-	0.1093	0.4951	52	0.1826	0.5242
Creatinine						
High	14	2.21 (1.25–3.92)	1.5 (0.87–2.59)	28	2.04 (1.06–3.95)	1.2 (0.6–2.4)
Normal	34	0.0017 *	0.1499	47	0.0182 *	0.6110
eGFR						
Low	24	1.76 (1.02–3.05)	1.24 (0.66–2.33)	41	1.29 (0.67–2.49)	0.96 (0.47–1.96)
Normal	-	0.0634	0.5088	45	0.4557	0.9055
del 17p/TP53						
Present	15	1.69 (0.78–3.68)	1.23 (0.82–1.84)	30	1.97 (0.68–5.71)	1.03 (0.6–1.78)
Absent	25	0.1191	0.3247	52	0.1106	0.9128
*No data: n = 32*						
t(4;14) IGH/FGFR3						
Present	42	0.87 (0.36–2.12)	1.32 (0.47–3.65)	-	1.26 (0.40–4.02)	1.52 (0.51–4.55)
Absent	24	0.7707	0.5994	46	0.6608	0.4514
*No data: n = 22*						
t(11;14) IGH/CCND1						
Present	9	1.61 (0.57–4.56)	1.56 (0.66–3.7)	46	1.08 (0.36–3.22)	1.16 (0.39–3.43)
Absent	25	0.2657	0.3166	45	0.8852	0.7950
*No data: n = 22*						
t(14;16) IGH/MAF						
Present	9	4.68 (0.07–315.0.4)	2.2 (0.29–17.08)	28	3.3 (0.1–113.16)	2.35 (0.3–18.53)
Absent	25	0.0872	0.4534	46	0.2082	0.4189
*No data: n = 22*						
Other IGH rearrangement						
Present	17	1.13 (0.48–2.64)	1.08 (0.48–2.46)	-	1.15 (0.38–3.51)	1.42 (0.48–4.19)
Absent	25	0.7620	0.8487	45	0.7936	0.5287
*No data: n = 22*						
*IL1B* genotype (rs1143634)						
CC	24	1.69 (1.01–2.84)	1.28 (0.72–2.27)	30	2.04 (1.1–3.78)	2.03 [1.06–3.88]
TT or TC	25	0.0424 *	0.4064	48	0.0184 *	0.0337 *
*IL1B* genotype (rs1143634)						
TT	-	0.60 (0.28–1.26)	0.76 (0.3–1.92)	-	0.14 (0.06–0.32)	0.16 (0.02–1.15)
TC or CC	24	0.2549	0.5695	38	0.0198 *	0.0700
IL-1β plasma level [pg/mL]						
Low	25	0.89 (0.53–1.5)	0.97 (0.57–1.66)	46	0.70 (0.38–1.31)	1.20 (0.64–2.23)
High	24	0.6655	0.9164	34	0.2507	0.5724

*—Statistically significant result. Abbreviations: aHSCT—autologous hematopoietic stem cell transplantation; B2M—beta-2-microglobulin; CI—confidence interval; CRP—C-reactive protein; CTD—cyclophosphamide, thalidomide, dexamethasone; eGFR—estimated glomerular filtration rate; HR—hazard ratio; IgA—immunoglobulin A; IgG—immunoglobulin G; IGH—immunoglobulin heavy chain; IL-1β—interleukin 1 beta; ISS—Multiple Myeloma International Staging System; LDH—lactate dehydrogenase; mOS—median overall survival; mPFS—median Progression-free survival; N/a—not applicable; WHO—World Health Organization; V(C)D—bortezomib, (cyclophosphamide), dexamethasone; VTD—bortezomib, thalidomide, dexamethasone.

## Data Availability

The data presented in this study are available in this article and Supplementary Materials.

## Supplementary Materials

The following supporting information can be downloaded at: https://www.mdpi.com/article/10.3390/cancers16071263/s1, Table S1: Distribution of selected demographic, clinical and molecular variables depending on gender; Table S2 Distribution of *IL1B* genotypes depending on selected demographic, clinical and molecular variables; Table S3: Comparisons of demographic and clinical variables depending on *IL1B* genotypes; Table S4: Comparisons of IL-1β concentration depending on selected demographic, clinical and molecular variables in the study group; Table S5: The correlation between demographic, and clinical variables and IL-1β level in the study group.
