# Supplementary material for: IL1B Polymorphism (rs1143634) and IL-1β Plasma Concentration as Predictors of Nutritional Disorders and Prognostic Factors in Multiple Myeloma Patients
Source: Cancers (Basel). 2024 Mar 24;16(7):1263. doi: 10.3390/cancers16071263 (PMC11011170; doi:10.3390/cancers16071263)
Supplement: Supplementary file 1 [file cancers-16-01263-s001.zip › cancers-2896138-supplementary.pdf]

Table S1. Distribution of selected demographic, clinical and molecular variables depending on gender.

Variable	Gender		
	**Female [n=49]**	**Male [n=44]**	***p***
**Age**			
≥65	30 (61.2%)	19 (43.2%)	0.1255
<65	19 (38.8%)	25 (56.8%)	
**Diagnosis**			0.8342
MM with a monoclonal component	43 (89.6%)	39 (86.7%)	
Light chain disease	5 (10.4%)	6 (13.3%)	
**Monoclonal protein class**			
IgG	27 (62.8%)	28 (71.8%)	0.5279
IgA	16 (37.2%)	11 (28.2%)	
N/a: n=11			
**Light chain type**			
Lambda	21 (42.8%)	15 (34.1%)	0.5135
Kappa	28 (99.6%)	29 (65.9%)	
**ISS stage**			
3	19 (38.8%)	16 (36.4%)	0.9798
1, 2	30 (61.2%)	28 (63.6%)	
**Renal function**			
B	6 (12.2%)	7 (15.9%)	0.8342
A	43 (87.3%)	37 (84.1%)	
**Stage of chronic kidney disease**			
G3a,G3b, G4, G5D	18 (36.7%)	15 (34.1%)	0.9609
G1,G2	31 (63.3%)	29 (65.9%)	
**Performance status**			
2-4	25 (51%)	24 (54.5%)	0.8950
0, 1	24 (49%)	20 (45.5%)	
**Treatment protocol (1)**			
CTD	10 (20.4%)	17 (38.6%)	0.0882
V(C)D, VTD	39 (79.6%)	27 (61.4%)	
**Treatment protocol (2)**			
VTD	13 (26.5%)	15 (34.1%)	0.5706
CTD, V(C)D	36 (73.5%)	29 (65.9%)	
**Anaemia before treatment (WHO)**			0.2970
Yes	7 (14.3%)	11 (25%)	
No	42 (85.7%)	33 (75%)	
**Platelets**			
Low	7 (14.3%)	5 (11.4%)	0.9125
Normal	42 (85.7%)	39 (88.6%)	
**Albumins**	14 (28.6%)	20 (45.5%)	0.1409

Low<br>Normal	35 (71.4%)	24 (54.5%)	
**CRP**<br>High<br>Normal	 14 (28.5%)<br>35 (71.5%)	 20 (45.4%)<br>24 (54.6%)	0.1409
**LDH**<br>High<br>Normal	 8 (19.5%)<br>41 (80.5%)	 2 (4.5%)<br>42 (95.5%)	0.1347
**Calcium**<br>High<br>Normal	 9 (22.5%)<br>40 (77.5%)	 12 (27.3%)<br>32 (72.7%)	0.4371
**B2M**<br>High<br>Normal	 40 (81.6%)<br>9 (18.4%)	 38 (86.4%)<br>6 (13.6%)	0.7361
**Creatinine**<br>High<br>Normal	 12 (24.5%)<br>37 (75.5%)	 20 (45.5%)<br>24 (54.5%)	0.0566
**eGFR**<br>Low<br>Normal	 31 (63.3%)<br>18 (36.7%)	 30 (68.2%)<br>14 (31.8%)	0.7797
**del 17p/TP53**<br>Present<br>Absent<br>*No data: n=32*	 8 (26.7%)<br>22 (73.3%)	 8 (25.8%)<br>23 (74.2%)	0.8300
**t(4;14) IGH/FGFR3**<br>Present<br>Absent<br>*No data: n=22*	 4 (11.1%)<br>32 (88.9%)	 6 (17.1%)<br>29 (82.9%)	0.6971
**t(11;14) IGH/CCND1**<br>Present<br>Absent<br>*No data: n=22*	 5 (13.9%)<br>31 (86.1%)	 3 (8.6%)<br>32 (91.4%)	0.7391
**t(14;16) IGH/MAF**<br>Present<br>Absent<br>*No data: n=22*	 0 (0%)<br>36 (100%)	 1 (2.8%)<br>34 (97.2%)	0.9887
**Other IGH rearrangement**<br>Present<br>Absent<br>*No data: n=22*	 9 (25%)<br>27 (75%)	 2 (5.4%)<br>33 (94.6%)	0.0552
***IL1B* genotype**<br>**(rs1143634)**<br>CC<br>TT or TC	 <br>22 (44.9%)<br>27 (55.1%)	 <br>29 (65.9%)<br>15 (34.1%)	0.0681

***IL1B* genotype (rs1143634)**<br>TT<br>TC or CC	 9 (18.4%)<br>40 (81.6%)	 4 (9.1%)<br>40 (90.9%)	0.3229
**IL-1β plasma level [pg/mL]**<br>Low<br>High	 26 (53.1%)<br>23 (46.9%)	 21 (47.7%)<br>23 (52.3%)	0.7596

* - statistically significant result

Abbreviations: B2M – beta-2-microglobulin, CRP – C-reactive protein, CTD—cyclophosphamide, thalidomide, dexamethasone, eGFR – estimated glomerular filtration rate, IgA – immunoglobulin A, IgG – immunoglobulin G,IGH – immunoglobulin heavy chain, IL-1β – interleukin 1 beta, ISS - Multiple Myeloma International Staging System, LDH - lactate dehydrogenase, WHO -World Health Organization, V(C)D—bortezomib, (cyclophosphamide), dexamethasone; VTD—bortezomib, thalidomide, dexamethasone.

Table S2. Distribution of *IL1B* genotypes depending on selected demographic, clinical and molecular variables.

Variable	*IL1B* genotypes			*p*
	CC	TT	TC	
**Gender**				
Men	29 (65.9%)	4 (9.1%)	11 (25%)	0.1155
Woman	22 (44.9%)	9 (18.4%)	18 (36.7%)	
**Diagnosis**				
MM with a monoclonal component	43 (87.7%)	12 (92.3%)	25 (86.2%)	0.6614
Light chain disease	6 (12.3%)	1 (7.7%)	4 (13.7%)	
**Monoclonal protein class**				
IgA	15 (32.6%)	6 (50%)	6 (25%)	0.3216
IgG	31 (67.4%)	6 (50%)	18 (75%)	
N/a: n=11				
**Light chain type**				
Lambda	19 (37.3%)	5 (38.5%)	12 (41.4%)	0.9357
Kappa	32 (62.71%)	8 (61.5%)	17 (58.6%)	
**ISS stage**				
3	18 (35.3%)	6 (46.2%)	12 (33.3%)	0.1458
2	21 (41.2%)	1 (7.7%)	7 (24.1%)	
1	12 (23.5%)	6 (46.2%)	10 (35.7%)	
**ISS stage**				
3	19 (37.3%)	6 (46.2%)	10 (34.4%)	0.7680
1, 2	32 (62.7%)	7 (53.8%)	19 (65.6%)	
**Renal function**				
B	8 (15.7%)	1 (7.7%)	4 (13.8%)	0.7589
A	43 (84.3%)	12 (92.3%)	25 (86.2%)	
**Stage of chronic kidney disease**				
G3a, G3b, G4, G5D	18 (35.3%)	5 (38.5%)	10 (34.5%)	0.9686
G1, G2	33 (64.7%)	8 (61.5%)	19 (65.5%)	
**Body weight loss before treatment**				
Yes	26 (56.5%)	7 (53.8%)	13 (44.8%)	0.8203
No	25 (53.2%)	6 (46.2%)	16 (55.2%)	
**Anaemia grade before treatment (WHO)**				
Absent or I°	43 (84.3%)	10 (76.9%)	22 (75.9%)	0.6126
II°, III° or IV°	8 (15.7%)	3 (23.1%)	7 (24.1%)	
**Haemoglobin**				
Low	45 (88.2%)	11 (84.6%)	24 (82.8%)	0.7843
Normal	6 (11.8%)	2 (15.4%)	5 (17.2%)	
**Platelets**				
Low	7 (13.7%)	1 (7.7%)	4 (13.8%)	0.8331
Normal	44 (86.3%)	12 (92.3%)	25 (86.2%)	

**Albumins**				
Low	23 (45.1%)	3 (23.1%)	8 (27.6%)	0.1629
Normal	28 (54.9%)	10 (76.9%)	21 (72.4%)	
**CRP**				
High	28 (54.9%)	2 (15.4%)	4 (13.8%)	0.0003*
Normal	23 (45.1%)	11 (84.6%)	25 (86.2 %)	
**LDH**				
High	5 (9.8%)	1 (17.7%)	4 (13.8%)	0.7969
Normal	46 (90.2%)	12 (92.3%)	25 (86.2%)	
**Calcium**				
High	13 (25.5%)	3 (23.1%)	5 (17.2%)	0.6971
Normal	38 (74.5%)	10 (76.9%)	24 (82.8%)	
**B2M**				
High	45 (88.2%)	11 (84.6%)	22 (75.9%)	0.3502
Normal	6 (11.8%)	2 (15.4%)	7 (24.1%)	
**Creatinine**				
High	20 (39.2%)	5 (38.5%)	7 (24.1%)	0.3730
Normal	31 (60.8%)	8 (61.5%)	22 (75.9%)	
**eGFR**				
Low	33 (64.7%)	8 (61.5%)	20 (69%)	0.8787
Normal	18 (35.3%)	5 (38.5%)	9 (31%)	
**del 17p/TP53**				
Absent	21 (65.6%)	8 (80%)	16 (84.2%)	0.3060
Present	11 (34.4%)	2 (20%)	3 (15.8%)	
No data: n=32				
**t(4;14) IGH/FGFR3**				
Absent	30 (83.3%)	10 (83.3%)	21 (91.3%)	0.6648
Present	6 (16.7%)	2 (16.7%)	2 (8.7%)	
No data: n=22				
**t(14;16) IGH/MAF**				
Absent	35 (97.2%)	12 (100%)	23 (100%)	0.6108
Present	1 (2.8%)	0 (0%)	0 (0%)	
No data: n=22				
**t(11;14) IGH/CCND1**				
Absent	32 (88.9%)	11 (91.7%)	20 (87%)	0.9154
Present	4 (11.1%)	1 (8.3%)	3 (13%)	
No data: n=22				
**Other IGH rearrangement**				
Absent	29 (80.5%)	11 (91.7%)	20 (86.9%)	0.6051
Present	7 (19.5%)	1 (8.3%)	3 (13.1%)	

No data: n=22				
**IL-1β plasma level [pg/mL]**<br>Low<br>High	 16 (31.4%)<br>35 (68.6%)	 10 (76.9%)<br>3 (23.1%)	 21 (72.4%)<br>8 (27.6%)	0.0002*

*—statistically significant results

Abbreviations: B2M – beta-2-microglobulin, CRP – C-reactive protein, eGFR – estimated glomerular filtration rate, IgA – immunoglobulin A, IgG – immunoglobulin G, IGH – immunoglobulin heavy chain, IL-1β – interleukin 1 beta, ISS - Multiple Myeloma International Staging System, LDH - lactate dehydrogenase, MM – Multiple Myeloma, N/a - not applicable, WHO -World Health Organization.

Table S3. Comparisons of demographic and clinical variables depending on *IL1B* genotypes.

Factor	*IL1B* genotype Median [IQR]					
	TT	CC and TC	*p*	CC	TT and TC	*p*
**BMI** [kg/m^2]	25.28 [21.99-27.20]	26.72 [23.95-29.4]	0.2612	26.77 [23.26-32]	25.31 [23.55-28.62]	0.2341
**Hgb** [g/dL]	10.9 [9.8-12.02]	9.95 [8.5-11.45]	0.1262	9.4 [8.35-11.4]	10.65 [9.2-11.8]	0.0526
**PLT** [K/μl]	213 [187.5-284.25]	204 [157-247]	0.3081	206 [157-254.5]	205.5 [159-234]	0.9415
**CRP** [mg/L]	1.75 [1.1-3.2]	3.8 [1.02-14.97]	0.2166	8.3 [1.4-28.4]	2 [1-4.1]	0.0014*
**LDH** [IU/L]	269.5 [243-407]	310 [267.25-382]	0.5760	310 [276-365.25]	307.5 [249-415]	0.8788
**Calcium** [mmol/L]	2.36 [2.27-2.49]	2.38 [2.25-2.49]	0.9074	2.4 [2.2-2.57]	2.36 [2.26-2.46]	0.8349
**B2M** [ng/L]	3.7 [2.87-8.59]	4.7 [2.72-7.88]	0.9503	4.77 [2.76-6.97]	4.33 [2.78-8.48]	0.8202
**Albumin** [g/dL]	3.91 [3.47-4.05]	3.60 [3.2-4]	0.2582	3.6 [3.02-3.81]	3.85 [3.4-4.2]	0.0165*
**Creatinine** [mg/dL]	0.78 [0.75-1.47]	0.98 [0.76-1.36]	0.5571	1.09 [0.81-1.47]	0.87 [0.74-1.3]	0.1566
**eGFR** [mL/min/1.73m^2]	78.2 [49.62-95]	75.88 [48.06-95]	0.8439	71.21 [45.26-95]	83.36 [53.06-95]	0.6269
**IL-1β plasma level** [pg/mL]	5.05 [4.59-6.02]	6.27 [4.79-12.28]	0.0567	7.56 [5.38-13.29]	4.97 [4.2-5.85]	<0.0001*

* — statistically significant results

Abbreviations: BMI – body mass index, B2M – beta-2-microglobulin, CRP – C-reactive protein, eGFR – estimated glomerular filtration rate, Hgb – hemoglobin, IL-1β – interleukin 1 beta, IQR– interquartile range, LDH - lactate dehydrogenase, PLT – platelets.

Table S4. Comparisons of IL-1β concentration depending on selected demographic, clinical and molecular variables in the study group.

**Variable**	**IL-1β plasma concentration** [pg/mL]	*p*
**Gender**		
Men	6.27	0.3284
Woman	5.65	
**Age**		
≥65	5.78	0.7729
<65	5.71	
**Diagnosis**		
Light chain disease,	5.85	0.6990
MM with a monoclonal component	5.71	
**Monoclonal protein class**		
IgA	5.52	0.5638
IgG	5.78	
**Light chain type**		
Lambda	6.94	0.7315
Kappa	5.65	
**ISS stage**		
3	5.61	0.4921
2	6.31	
1	5.38	
**ISS stage**		
3	6.31	0.4729
1, 2	5.71	
**Renal function**		
B	5.39	0.7207
A	5.65	
**Stage of chronic kidney disease**		
G3a, G3b, G4, G5D	5.58	0.8819
G1, G2	5.65	
**Body weight loss before treatment**		
Yes	6.50	0.2048
No	5.38	
**Anaemia grade before treatment(WHO)**		
Absent or I°	5.78	0.8041
II°, III° or IV°	7.46	
**Haemoglobin**		
Low	5.71	0.6416
Normal	9.34	

**Platelets**		
Low	6.33	0.8815
Normal	5.65	
**Albumins**		
Low	6.82	0.0752
Normal	5.52	
**CRP**		
High	8.35	0.0004*
Normal	5.38	
**LDH**		
High	5.75	0.7191
Normal	5.78	
**Calcium**		
High	5.78	0.9304
Normal	5.71	
**B2M**		
High	6.01	0.0857
Normal	5.12	
**Creatinine**		
High	5.81	0.8779
Normal	5.65	
**eGFR**		
Low	5.78	0.9516
Normal	5.98	
**del 17p/TP53**		
Absent	5.52	0.1135
Present	6.31	
No data: n=32		
**t(4;14) IGH/FGFR3**		
Absent	5.65	0.6434
Present	8.58	
No data: n=22		
**t(14;16) IGH/MAF**		
Absent	5.78	0.4944
Present	5.08	
No data: n=22		
**t(11;14) IGH/CCND1**		
Absent	5.78	0.5667
Present	5.41	
No data: n=22		

**Other IGH rearrangement**<br>Absent<br>Present<br>*No data: n=22*	 5.62<br>8.02	0.3090

*—statistically significant results

Abbreviations: B2M – beta-2-microglobulin, CRP – C-reactive protein, eGFR – estimated glomerular filtration rate, IgA – immunoglobulin A, IgG – immunoglobulin G, IGH – immunoglobulin heavy chain, IL-1β – interleukin 1 beta, ISS - Multiple Myeloma International Staging System, LDH - lactate dehydrogenase, MM – Multiple Myeloma, WHO – World Health Organization.

Table S5. The correlation between demographic, and clinical variables and IL-1β level in the study group.

**Factor**	**IL-1β plasma concentration** [pg/mL]	
	**r**	***p***
**Age** [years]	-0.012	0.9093
**BMI** [kg/m^2]	0.132	0.2410
**ISS stage**	0.051	0.5854
**Stage of chronic kidney disease**	0.043	0.6284
**Hgb** [g/dL]	-0.117	0.2656
**Anaemia grade before treatment (WHO)**	0.323	0.2558
**PLT** [K/µl]	0.093	0.3730
**CRP** [mg/L]	0.295	0.0045*
**LDH** [IU/L]	0.014	0.9013
**Calcium** [mmol/L]	-0.050	0.6320
**B2M** [ng/L]	0.073	0.4931
**Albumin** [g/dL]	-0.180	0.0836
**Creatinine** [mg/dL]	0.078	0.4588
**eGFR** [mL/min/1.73 m^2]	-0.051	0.6246

*—statistically significant results

Abbreviations: BMI – body mass index, B2M – beta-2-microglobulin, CRP – C-reactive protein, eGFR – estimated glomerular filtration rate, Hgb – hemoglobin, IL-1β – interleukin 1 beta, ISS – Multiple Myeloma International Staging System, LDH - lactate dehydrogenase, PLT – platelets, WHO -World Health Organization.
